# Lateralization of Temporal Lobe Epilepsy Based on Resting-State Functional Magnetic Resonance Imaging and Machine Learning

**DOI:** 10.3389/fneur.2015.00184

**Published:** 2015-08-31

**Authors:** Zhengyi Yang, Jeiran Choupan, David Reutens, Julia Hocking

**Affiliations:** ^1^School of Information Technology and Electrical Engineering, The University of Queensland, Brisbane, QLD, Australia; ^2^Centre for Advanced Imaging, The University of Queensland, Brisbane, QLD, Australia; ^3^Queensland Brain Institute, The University of Queensland, Brisbane, QLD, Australia; ^4^School of Psychology and Counselling, Queensland University of Technology, Brisbane, QLD, Australia

**Keywords:** temporal lobe epilepsy, laterality of TLE, resting-state functional connectivity, machine learning, feature selection

## Abstract

Lateralization of temporal lobe epilepsy (TLE) is critical for successful outcome of surgery to relieve seizures. TLE affects brain regions beyond the temporal lobes and has been associated with aberrant brain networks, based on evidence from functional magnetic resonance imaging. We present here a machine learning-based method for determining the laterality of TLE, using features extracted from resting-state functional connectivity of the brain. A comprehensive feature space was constructed to include network properties within local brain regions, between brain regions, and across the whole network. Feature selection was performed based on random forest and a support vector machine was employed to train a linear model to predict the laterality of TLE on unseen patients. A leave-one-patient-out cross validation was carried out on 12 patients and a prediction accuracy of 83% was achieved. The importance of selected features was analyzed to demonstrate the contribution of resting-state connectivity attributes at voxel, region, and network levels to TLE lateralization.

## Introduction

Surgical intervention is the treatment of choice for controlling seizures in patients with epilepsy refractory to medication. Relief from seizures has been shown in 70% of patients with focal epilepsies, with the most positive outcome observed in temporal lobe epilepsy (TLE) (1). Lateralization and localization of the locus of seizure are therefore a critical component of pre-surgical evaluation in patients with TLE (2).

Evidence for altered functional connectivity (FC) and changes to the default mode network (DMN) in patients with TLE has been reported using resting-state functional magnetic resonance imaging (rfMRI) (3–10). For example, Pereira and colleagues found asymmetrical hippocampal connectivity in mesial TLE patients (11), and reduced connectivity in the posterior (retrosplenium/precuneus) to anterior (ventromedial pre-frontal cortex) DMN in patients with TLE was reported by Haneef et al. (5). These DMN characteristics have been utilized to define support vector machine (SVM) features, including global connectivity asymmetry and pair-wise brain region synchronization. In one report, this technique has resulted in an accuracy level of 83.9% for distinguishing patients with epilepsy from healthy controls (12). A computer-aided diagnosis tool based on FDG–PET was reported to have accuracy of 82 and 88% in distinguishing left TLE and right TLE from non-seizures, respectively. The classifier that both diagnosed and lateralized the disease had overall accuracy of 76%, where 89% of patients correctly identified with epilepsy were correctly lateralized (13).

Morgan et al. identified that a network involving the right hippocampus and right thalamus can be used to categorize patients into left or right TLE (2). Reduced posterior DMN connectivity in a group of patients with right TLE contrasted with increased connectivity in the posterior and anterior DMN in a group of patients with left TLE has also been reported (5). In a study on lesion-negative TLE patients, an individual laterality index was used to determine seizure lateralization, and found that 88% of cases agreed with the clinical diagnosis (14). In addition to pair-wise FC among spatially segregated brain regions, local network properties have also been explored to localize TLE. In a cohort of children with TLE, increased Regional Homogeneity (ReHo) in the posterior cingulate gyrus and the right medial temporal lobe was uncovered (15). Increased amplitude of low-frequency fluctuation (ALFF) in the mesial temporal lobe and thalamus, decreased ALFF in regions of the DMN, altered network topological properties, and causal connectivity have been found in mesial TLE patients (16–18). In four patients with focal TLE, ReHo combined with an intra-regional connectivity defined as the ratio of the mean pair-wise correlations of all voxels within a region of interest (ROI) with the corresponding contralateral region was used to select the epileptogenic zone from a set of anatomically defined ROIs (19). We have previously identified brain regions with significantly different FC, ReHo, or ALFF between left and right TLE groups (20).

Based on these informative findings, the aim of the present study was to test the hypothesis that resting-state FC and network characteristics might be useful for lateralization of TLE, providing complementary information to other clinical diagnostic measures. We formulated the lateralization of TLE based on rfMRI as a supervised machine learning problem. We constructed a comprehensive feature space to include quantities that may improve the localization of seizure foci. Feature selection was carried out to deal with the “curse of dimensionality” and a leave-one-out cross validation (LOOCV) was employed to train an SVM model and test its performance. Feature importance analysis was conducted to identify features or combinations of features that were informative to TLE lateralization.

## Materials and Methods

### Participants

Twelve pre-surgical patients with unilateral left or right TLE took part in the study. Seven patients had left TLE and five had right TLE. Table 1 shows the demographic characteristics, clinical ratings, and Wechsler Adult Intelligence Scale, third edition test scores for participants. Groups were matched for age, onset age, and intelligence scores. We note that all left TLE patients are male. Subject-level demographics can be found in Supplementary Material. There was no involvement of extratemporal structures, based on clinical, electrographic, and neuroimaging assessments carried out at the Neurology Department of the Royal Brisbane and Women’s Hospital (RBWH), QLD, Australia. The study was approved by the RBWH Research Ethics Committee and The University of Queensland Medical Research Ethics Committee. Written informed consent was obtained prior to scanning from each patient.

**Table 1:** **Participant characteristics**.

Characteristic	Left TLE	Right TLE	*p* Value*
**No. of participants**	7	5	
**Age**, mean (SD) [range] (years)	38 (11) [22–54]	33 (13) [22–56]	0.41
**Gender**			
Male	4	0	0.08
Female	3	5	
**Onset Age**, mean (SD) [range] {No. of valid entries} (years)	31 (15) [18–47] {3}	8 (9) [0.5–19] {4}	0.11
**WAIS-III**, mean (SD) [range] {No. of valid entries}			
VIQ	49 (18) [34–73] {4}	62 (16) [44–75] {5}	0.29
PIQ	49 (12) [38–62] {4}	43 (4) [39–49] {5}	0.92
FSIQ	98 (29) [73–135] {4}	105 (18) [84–121] {5}	0.56

*TLE, temporal lobe epilepsy; WAIS, Wechsler adult intelligence scale; VIQ, verbal IQ; PIQ, performance IQ; FSIQ, full scale IQ*.

*Calculated using Mann–Whitney U tests to compare the groups for age, onset age, and WAIS-III scores, and Fisher’s exact test to compare the groups by gender.

### Data acquisition

All MRI images were acquired on a Siemens Trio^®^ 3-T scanner. The resting-state scan comprised one component of a larger functional imaging study, for which patients underwent one resting-state and four task-based functional runs, and one T1-weighted structural scan. The resting-state scan was the final set of data acquired, with duration of 6 min, and patients were instructed to lie still with their eyes closed. Functional images used a T2*-weighted EPI sequence for blood oxygen level dependent (BOLD) contrast. Imaging parameters were TR/TE 2500/34 ms, flip angle 90°, 36 slices with acquisition matrix 64 × 64, field of view 260 mm × 260 mm, slice thickness 3.0 mm, and reconstructed voxel size 3.3 mm × 3.3 mm × 3.3 mm.

### Image preprocessing

DPARSFA (21) and REST (22) software were employed for fMRI data processing. The image volumes at the first several time points were removed to allow patient adaptation and signal stabilization, resulting in 135 volumes of each patient retained for further analysis. The time difference between slices was corrected and scans were checked for excessive head motion (larger than 3 mm or 3°). The images were realigned to the middle slice and spatially normalized to the MNI template (61 × 73 × 61, isotropic voxel size of 3 mm). A Gaussian smoothing kernel with a full-width at half-maximum (FWHM) of 4 mm was applied, followed by linear detrending and bandpass filtering (0.01–0.08 Hz).

Using the automated anatomical labeling (AAL) atlas (23), the brain was parcellated into 116 regions, including 90 regions in the cerebra (45 in each hemisphere) and 26 regions in the cerebella (9 in each cerebellar hemisphere and 8 in the vermis). These ROIs were used as nodes for constructing the resting-state functional network.

### Feature space construction

In this study, features are informative attributes derived from MRI data in discriminating left TLE from right TLE. We included the following three categories of measurements to form a bag of candidate features, from which important features were selected for machine learning using SVM:

(1) Univariate features: these voxelwise features reflect the local properties of resting-state brain activity at voxel level, including ALFF, fractional ALFF (fALFF), and ReHo. ALFF measures the regional spontaneous activities and it was found being significantly larger than the global mean ALFF in vicinity of large blood vessels (24). To overcome the issue of ALFF being sensitive to physiological noise, fALFF was proposed as the ratio between the total amplitude with low-frequency range (typically 0.01–0.08 Hz) to the total amplitude of the entire detectable frequency range (25). Unlike measuring the signal synchrony of low-frequency fluctuation activities in different parts of the brain, ReHo is defined as the dependence of the resting-state time course of a given voxel with those of its immediate neighbors (26). It thus quantifies the intra-regional connectivity. ReHo was calculated using Kendall’s coefficient of concordance (KCC) with 26 neighboring voxels and then smoothed (FWHM = 4 mm). The individual ReHo, ALFF, and fALFF maps were divided by the corresponding patient-specific global mean values for standardization purpose. ALFF and fALFF were computed on data before bandpass filtering. ReHo was calculated on unsmoothed data.
(2) Bivariate features: these features describe the pair-wise connectivity between brain regions, or inter-regional connectivity. For each cerebral region, time courses were extracted and averaged over the ROIs defined in the AAL atlas. Several nuisance covariates associated with physiological processes were regressed out, including the estimated head-motion parameters, whole brain signal, white matter (WM) signal, and cerebrospinal fluid (CSF) signal. We used the default masks in REST for regressing out the WM and CSF signals. The default masks were made from the *a priori* templates found in SPM as follows (22): the whole brain mask was from brainmask.nii with a threshold at 50% probability, the WM mask was from white.nii with a threshold at 90% probability, and the CSF mask was from csf.nii with a threshold at 70% probability. The inter-regional connectivity was computed using Pearson’s correlation coefficient, resulting in an FC matrix with 116 × 116 entries. To improve the normality of the coefficients, a Fisher’s *z* transformation was applied. For each FC, one-sample *t*-test against 0 was performed and FCs survived the test were taken as candidate bivariate features.
(3) Multivariate features: these features, referring to the global and nodal metrics of whole brain resting-state network, were computed using the Brainnetome Toolkit (27). Individual FC matrix was binarized to have entries indicating whether connectivity exists between any two given regions. Different threshold values result in different levels of connectivity density. To cover a wide range of density levels and enable the calculation of small-worldness (28), six threshold values from 0.05 to 0.3 with a step of 0.05 were used for binarizing the FC matrices. A binarized matrix represents a graph and two categories of graph theory-based network metrics were calculated (29, 30): (i) metrics defined for both the whole network (network-wide) and each node (nodal), including degree, shortest path length, global efficiency, local efficiency, and clustering coefficient. (ii) Metrics defined for the whole network only, including assortativity (31), transitivity (32), and small-worldness (33). Therefore, two sub-categories of multivariate features were included as candidate features: global network metrics (NMglobal) and nodal network metrics (NMnodal). Brief descriptions of the network metrics can be found in Table 5 in the Results Section.

Therefore, we had six sub-categories of features: ALFF, fALFF, ReHo, FC, NMglobal, and NMnodal.

### Group comparison

As references of feature importance, significant group differences in the features were identified using Mann–Whitney *U*-test (*p* < 0.01). For ALFF, fALFF, and ReHo, multiple comparison errors were corrected using the AlphaSim method (34) (6-connection clusters, cluster size ≥16 voxels, i.e., 432 mm^3^, *p* < 0.01).

### Classifier training and testing

Support vector machine is the most widely used classification method for multivariate fMRI analysis (35). In this study, we trained SVM models with linear kernels using LIBSVM toolbox (36). In each LOOCV run, one patient was left out as “unseen” test data and the remaining 11 subjects’ data were used for feature selection and SVM model training. The performance of the trained classifiers was evaluated using correct rate, sensitivity, and specificity.

### Feature selection and feature importance

The constructed feature space contains thousands of candidate features. Problem of model over-fitting, i.e., the “curse of dimensionality,” would occur if all of them were used for training a classifier. Random forest (RF) (37) was used to select features in this study. RF is a random ensemble of decision trees and has intrinsic advantages in dealing with the “curse of dimensionality.” In RF, every time a split of a node is made on a given feature the Gini impurity criterion for the two descendent nodes is less than the parent node. Feature importance of an individual feature was estimated by adding up the decreases in Gini impurity over all trees in the forest.

We adopted a feature selection strategy involving a ranking of explanatory variables using RF (38). In each LOOCV run, feature importance calculation was repeated 50 times for each category separately, and the features in each sub-category were ranked by their average importance. The top 50 features from each sub-category were pooled to form a feature set with 300 features and ranked again. A collection of RF models were trained by adding features from the most important to the least important one by one. The minimum feature set leading to the smallest out-of-bag (OOB) error rate was selected. Note that each LOOCV run might have different numbers of final features.

To analyze the contribution of each sub-category of features to the lateralization of TLE, we evaluated the importance of each feature according to its rank and occurrence in the 12 LOOCV runs. In each run, the most important selected feature was assigned the maximum score, which is the number of total features selected in the run, while the least importance one was assigned a score of 1. Then, the scores in each run were normalized by dividing the total score of that run. Feature-specific and sub-category-specific importance was then calculated as the summation of relevant normalized scores.

## Results

All subjects had translational head motion less than one voxel length (3 mm) and rotational motion <3° in the scan session and were included in the analyses. The left TLE group had a larger mean value of the maximum translational motion along all three axes than the right TLE group. The group mean of the maximum translational motion along the *z*-axis was the largest in the three axes in both left TLE group (0.73 ± 0.45 mm, along *z*-axis) and the right TLE group (0.46 ± 0.26 mm). No significant differences in the median values of the six motion parameters were found (Mann–Whitney *U*-test, *p* values were 0.43, 0.79, 0.43, 0.25, 1.00, and 0.33 for the three translational and the three rotational motion, respectively).

### Classification performance

The SVM classifier trained on the final feature set achieved 83.33% correct rate in the 12 cross validation runs. The results of the 12 runs are shown in Table 2. The sensitivity and specificity to the left TLE was 0.86 and 0.80, respectively.

**Table 2:** **Classification results and the numbers of features selected for the 12 LOOCV runs**.

I D	1	2	3	4	5	6	7	8	9	10	11	12
Diagnos ed	L	L	L	L	L	R	R	R	R	L	R	L
Guessed	L	L	L	R	L	L	R	R	R	L	R	L
No. of features	13	1	9	4	11	6	25	14	9	9	9	13

*The correct rate is 0.83. Taking the left TLE as positive label, sensitivity is 0.86 and specificity is 0.8*.

*The gray shades indicate the two misclassified subjects*.

### Selected features

There were 54,837 candidate features of ALFF, fALFF, and ReHo. The numbers for FC, NMGlobal, and NMnodal were 1785, 66, and 4176, respectively. In the final selected 123 features of the 12 runs, there were 118 unique ones. The average number of selected feature per run was 10.25, ranging from 1 to 25.

**Figure 1:**
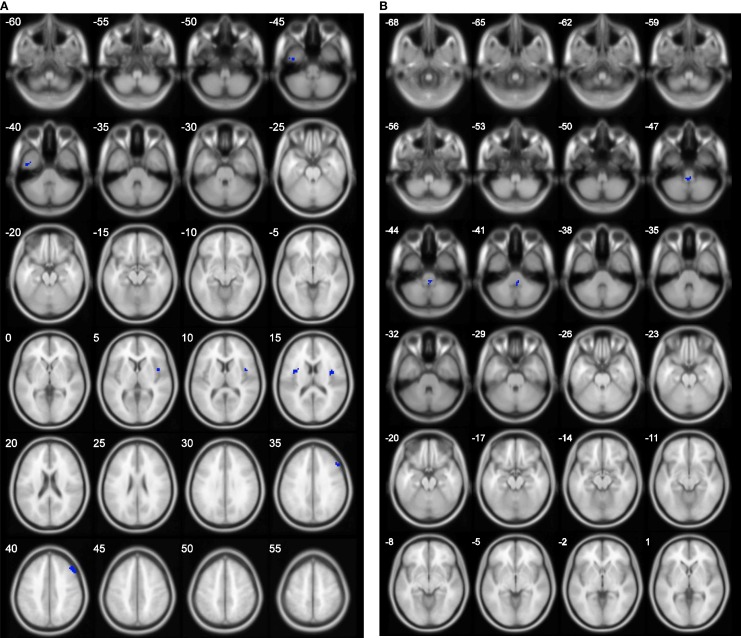
**Clusters with group difference in voxelwise properties of resting-state connectivity plotted in blue using xjView**. **(A)** ReHo with four clusters and **(B)** fALFF with one cluster. No cluster was found in ALFF.

The results of group comparison showed no region with group-wise difference in ALFF. The clusters with significant group difference in fALFF and ReHo are plotted in Figure 1 using xjView (http://www.alivelearn.net/xjview8/). The AAL ROIs containing these clusters are listed in Table 3. The AAL ROIs, MNI coordinates and scores of relative importance of the top five ranked selected features of ALFF, fALFF, and ReHo are in Table 4. Note that only 1 out of the 15 top ranked features was in AAL ROIs that had group difference, which was ReHo of a voxel in right middle frontal gyrus.Ho.

**Table 3:** **Regions with significant differences in fALFF and ReHo between the two groups**.

Measure	AAL regions (Tzourio-Mazoyer ID)	MNI	*N*
fALFF	Cerebelum_9_R(106)	0 −42 −48	17
ReHo	Insula_R(30), Rolandic_Oper_R(18)	45 6 3	36
	Insula_L(29), Rolandic_Oper_L(17)	−36 3 12	21
	Frontal_Mid_R(8)	54 24 36	24
	Temporal_Inf_L (89)	−39 −6 −48	22

*MNI: the coordinates of the voxel with peak U-test statistic in the cluster; N: the number of voxels in the cluster*.

**Table 4:** **Top five ranked voxels in ALFF, fALFF, and ReHo**.

Measure	AAL regions	MNI	Score
ALFF	Temporal_Inf_L	−57 −60 −9	1.00
	Parietal_Inf_L	−27 −69 42	0.23
	Frontal_Med_Orb_R	9 48 −12	0.17
	Cerebelum_7b_L	−39 −45 −42	0.12
	Temporal_Mid_R	48 −3 −24	0.11
fALFF	Middle Frontal Gyrus	45 30 45	0.24
	Frontal_Mid_L	−24 12 60	0.13
	Frontal_Sup_R	27 3 60	0.11
	Rectus_L	−3 27 −18	0.11
	Frontal_Inf_Tri_L	−54 33 15	0.10
ReHo	Vermis_6	0 −69 −24	0.27
	Cerebelum_8_R	27 −42 −51	0.18
	Cerebelum_8_R	39 −45 −54	0.15
	Temporal_Sup_R	69 −24 0	0.13
	Frontal_Mid_R	45 33 42	0.13

*MNI: the coordinates of the peak in the cluster; score: the normalized score indicating the relative features importance*.

**Figure 2:**
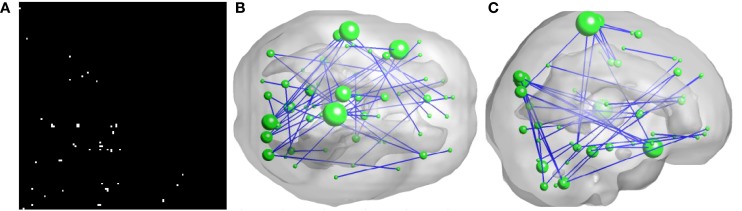
**Inter-regional resting-state functional connectivity**. **(A)** shows the matrix of which the entries indicate FCs with significant group difference (*U*-test, *p* < 0.01) A 3D rendering of the FCs, 50 in total, is shown on **(B,C)**. The diameter of a node is proportional to the number of identified FCs involving that node and the top five nodes are: right paracentral lobule (degree = 6), left superior temporal gyrus (degree = 5), left superior temporal pole (degree = 5), left paracentral lobule (degree = 4), and right cuneus (degree = 4).

**Figure 3:**
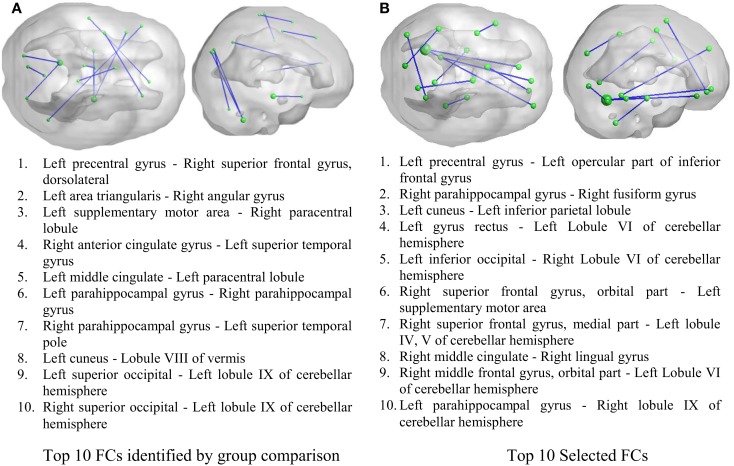
**Inter-regional resting-state functional connectivity**. **(A)** The top 10 FCs with smallest *p* value in group comparison. **(B)** The top 10 FCs selected by RF as features. Top: 3D rendering demonstrating the FCs. The nodal size is proportional to the nodal degree. Bottom: the AAL ROI names of the identified regions.

As illustrated in Figure 2, 50 FCs demonstrated significant between-group differences. The top 10 FCs with significant between-group difference and the top 10 selected FCs are shown in Figure 3. It is noted that there was no overlap between the two sets of FCs.

There were 66 global network metrics calculated, 11 at each of the 6 network density levels. For each AAL ROI, 6 nodal network metrics were computed at each network density level, resulting in 4176 candidate features. In the global network metrics, significant group differences were found in Gamma (threshold = 0.25) and shortest path length (threshold = 0.1 and 0.15). Compared with the most informative global network metrics listed in Table 5, both Gamma at threshold of 0.25 and shortest path length at threshold of 0.10 were selected as features.

**Table 5:** **Selected global and nodal network metric features**.

	NMglobal		NMnodal		
	*t*	Name	*t*	ROI	Name
1	0.20	Shortest path length	0.30	Right globus pallidus	Clustering coefficient
2	0.15	Assortivity	0.30	Left crus I of cerebellar hemisphere	Clustering coefficient
3	0.15	Lambda	0.15	**Left middle temporal pole**	**Local efficiency**
4	0.30	Small-worldness	0.25	Right cuneus	Shortest path length
5	0.25	Shortest path length	0.05	Lobule X of vermis (nodulus)	Degree
6	0.10	Clustering coefficient	0.10	Left orbital part of inferior frontal gyrus	Shortest path length
7	**0.10**	**Shortest path length**	0.25	Left middle frontal gyrus, orbital part	Shortest path length
8	0.15	Gamma	0.05	**Left hippocampus**	**Shortest path length**
9	0.20	Degree	0.10	Right superior occipital	Shortest path length
10	**0.25**	**Gamma**	0.05	Lobule X of vermis (nodulus)	Shortest path length

*The metric in bold indicates that it has been identified by group comparison as well*.

**t* is the threshold used for matrix binarization*.

Degree: the number of connections linked directly to a node

Neighbour degree: the average degree of the neighbours of a node

Global efficiency: the global efficiency of information propagation in the network

Local efficiency: the efficiency of information propagation through the direct neighbours of a node

Clustering coefficient: the extent of the local density or cliquishness of the network

Shortest path length: the extent of average connectivity or overall routing efficiency of the network

Gamma: the ratio between the extent of local clustering of a network and the surrogate random networks

Lambda: the ratio between the extent of overall routing efficiency of a network and the surrogate random networks

Smallworldness: the extent of a network between randomness and order

Assortativity: a bias in favour of connections between network nodes with similar characteristics

*Transivity: the fraction of triple-nodes that have their third edge filled in to complete the triangle*.

Sixty nodal network metrics in 22 AAL regions had group difference at various network density levels. Among them, seven regions demonstrated group differences when at least two different threshold values were used: left superior frontal gyrus in degree, global efficiency, and shortest path length; left hippocampus in degree, clustering coefficient, global efficiency, local efficiency, and shortest path length; right medial orbitofrontal cortex in global efficiency; left parahippocampal gyrus in clustering coefficient; left middle temporal pole in clustering coefficient and local efficiency; right middle temporal pole in clustering coefficient; lobule X of vermis in global efficiency and shortest path length. As shown in Table 5, only the local efficiency of the left middle temporal gyrus and the shortest path length of the left hippocampus were ranked in the top 10 category-specific informative features by RF.

**Figure 4:**
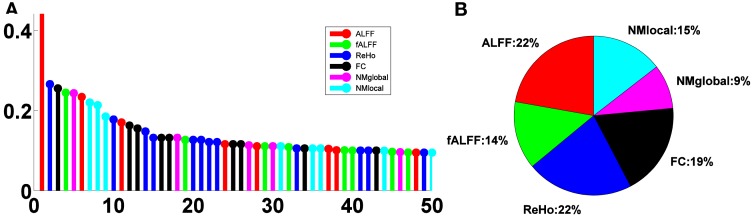
**Feature sub-category importance**. **(A)** Relative ranking of the top 50 features. **(B)** Relative importance of feature sub-categories.

The relative importance scores of the top 50 selected features are shown in Figure 4A. We note the largest one corresponding to the ALFF feature at a voxel at the left inferior temporal lobe selected in the second LOOCV run, where it was the only feature selected, thus having a score of 1. There were 18, 19, 29, 24, 11, and 17 features selected from ALFF, fALFF, ReHo, FC, NMglobal, and NMnodal, respectively. The percentage of the sub-category-specific contribution to the classification is shown in the pie chart of Figure 4, with ReHo and ALFF being the most (22% each) and the global network metrics the least (9%).

## Discussion

There is convergent evidence from fMRI and EEG studies supporting brains networks underlying the core phenomena in epilepsy, from seizure generation, cognitive dysfunction to response to treatment (39). In this study, we developed a method for predicting TLE lateralization based on a comprehensive feature space and 83% correct rate was achieved in a setting of LOOCV on 12 patients. The feature space was constructed to include information about intra-regional, inter-regional, and network-wide connectivities derived from resting-state fMRI. To deal with the problem of over-fitting, an efficient feature selection method was developed based on RF. Feature importance analysis revealed that global network metrics are less informative than inter- or intra-regional connectivities in TLE lateralization.

The discrepancies between the group-wise different connectivities and the top ranked features are obvious. We note the intrinsic difference between the two methods: the former is based on univariate analysis, while the latter is a multivariate method *per se*, because the feature importance was estimated by the joint contribution of a set of features to the prediction accuracy.

It is interesting, but not surprising, that the selected features are from multiple sub-categories in most LOOCV runs. The sub-category-specific important scores ranged from 9 to 22%. This may be reflecting that the brain network characterization of TLE laterality spans at different levels, from voxel, inter-regions, and brain-wide. More sophisticated kernel functions might be able to achieve higher prediction accuracy, but due to the small sample size of the study, to prevent over-fitting we employed the widely used linear kernel.

Morgan et al. identified a region in the ventral lateral nucleus of the right thalamus whose resting-state FC to the hippocampi separates left from right TLE patients (2). In the study on a cohort of seven seizure-free left TLE and seven seizure-free right TLE patients, a cut-off value of the mean connectivity between the right hippocampus and a small region in the right thalamus was found to be practicable for the lateralization of seizure-free TLE. Nevertheless, the cut-off value was not determined in a LOOCV setting. The performance of this method on unseen patients is still unknown. In our study, we did not find any significant group difference in the FC between the right thalamus and the right hippocampus. In the left TLE group, the right thalamus was found to have significant connectivity with six AAL regions, including the right insula, the left superior occipital, the right putamen, the left and the right globus pallidus, and the left thalamus, while the right hippocampus was significantly connected to the left hippocampus only. In the right TLE group, significant connectivity between the right thalamus and three regions, the right middle cingulate, the right caudate nucleus, and the left thalamus, were found, while the right hippocampus was significantly connected to the left hippocampus and the right dorsolateral superior frontal gyrus. The inconsistent results of the two studies with similar sample size might be attributed to the differences in FC calculations and cohorts. In Morgan’s study, the whole brain hippocampal FC was focused, which was calculated as whole brain voxelwise connectivity maps using the left and the right hippocampi as seed regions, respectively. In this study, we were interested in inter-regional connectivities between cerebral regions as predefined in AAL template, to avoid dealing with the much higher dimensionality of whole brain connectivity maps. It is possible, however, to include the connectivity identified in Morgan’s study as a promising feature in our framework for lateralization of TLE in future work. The inconsistency between the results also highlights the necessity of a large dataset to be used for rigorous validation.

Although the classification performance of this study using resting-state FC is promising, we note that there exist limitations related to sample size. A large independent data set is needed to further validate the proposed method and confirm the findings. Respiration and cardiac cycle-induced noise (40) were not considered because the required data were not available. Group differences in fALFF, but not ALFF, were found in this study, which contradicts the results of a pilot study (20) on a subset of the cohort. The regions with significant group difference in ReHo were not the same as in the pilot study. This can be partially explained by the different sample sizes, different statistical tests, and different multiple comparison correction criteria used in the two studies, as well as the inter-subject reproducibility issue of ALFF (25). We postulate that a large dataset is required to elucidate the reasons behind the variations.

The current study was aimed at the lateralization of TLE, which was solved as a binary classification problem. However, the proposed method has the potential to predict the loci of seizures at a finer scale, which can be formulated as a multi-class classification task. To do so, a large dataset with sufficient number of patients with TLE in different loci is needed. The characteristics of the resting-state FC, intra- and inter-regional connections as identified in this study, in particular, of patients with TLE in each brain region can be learned using the proposed method. The approach to constructing a comprehensive feature space with the ability to extract a wide range of information and subsequent feature selection method might be applicable to the investigations of other diseases based on resting-state fMRI.

## Conclusion

We presented an approach to lateralization of TLE based on resting-state fMRI scans. The approach relied on a feature set integrating the information about laterality encoded in intra-regional, inter-regional, and whole brain network connectivities to achieve 83% correct rate on a small cohort. RF-based feature selection, along with relative feature importance analysis, provides a multivariate analysis method for characterizing TLE laterality. Given the advantage of resting-state fMRI in terms of patient tolerance, the proposed approach can be a potential pre-surgical tool in future clinical practice, if validated in a larger independent cohort.

## Conflict of Interest Statement

The authors declare that the research was conducted in the absence of any commercial or financial relationships that could be construed as a potential conflict of interest.

## Supplementary Material

The Supplementary Material for this article can be found online at http://journal.frontiersin.org/article/10.3389/fneur.2015.00184
