# Supplementary material for: Lateralization of Temporal Lobe Epilepsy Based on Resting-State Functional Magnetic Resonance Imaging and Machine Learning
Source: Front Neurol. 2015 Aug 31;6:184. doi: 10.3389/fneur.2015.00184 (PMC4553409; doi:10.3389/fneur.2015.00184)
Supplement: Supplementary file 1 [file data_sheet_1.zip › 110661_TLE_suppl_1.docx]

**Supplementary Material 1.**

**Subject-level demographic and epilepsy-related characteristics.**

| **ID** | **Age** | **Gender** | **TLE Laterality** | **Onset Age** | **Seizure Type** | **MRI Findings** | **Routine EEG (Y/N)** | **Diffuse Background (Y/N)** | **Focal**  **(Y/N)** | **Spikes**  **(Y/N)** | **WAIS III VIQ** | **WAIS III PIQ** | **WAIS III FSIQ** | **Name of Medications** |
| --- | --- | --- | --- | --- | --- | --- | --- | --- | --- | --- | --- | --- | --- | --- |
| **01** | 49 | F | L | 47 | CPS | Hippocampal sclerosis | Y | N | N | N | 51 | 56 | 107 | Carbamazepine |
| **02** | 37 | M | L | 18 | CPS | Hippocampal sclerosis | **N/A** | **N/A** | **N/A** | **N/A** | 37 | 38 | 75 | Topiramate, Clonazepam, Carbamazepine |
| **03** | 45 | F | L | 27 | CPS | Incidental findings | Y | Y | Temporal Left | N | 73 | 62 | 135 | Phenytoin, Lamotrigine, Levitericitam |
| **04** | 22 | M | L | **N/A** | **N/A** | Left hippocampal sclerosis | **N/A** | **N/A** | **N/A** | **N/A** | **N/A** | **N/A** | **N/A** | **N/A** |
| **05** | 29 | M | L | **N/A** | **N/A** | Possible dysplasia or low grade tumour in left mesial temporal | **N/A** | **N/A** | **N/A** | **N/A** | **N/A** | **N/A** | **N/A** | **N/A** |
| **06** | 31 | F | R | 12 months | CPS and GTCS | Hippocampal sclerosis and possible cortical dysplasia | Y | N | Temporal Right | N | 44 | 43 | 87 | Carbamazepine, Levitericitam |
| **07** | 56 | F | R | 19 | CPS and SPS | Hippocampal sclerosis and gliosis (depth electrodes) | Y | N | N | Temporal Right and Left | 45 | 39 | 84 | Levetiracetam, Lamotrigine, Lacosimide, Atorvastatin,  Citalopram |
| **08** | 29 | F | R | **N/A** | **N/A** | Possible right hippocampal lesion / dysplasia | **N/A** | **N/A** | **N/A** | **N/A** | 72 | 49 | 121 | **N/A** |
| **09** | 22 | F | R | 6 months | CPS | Hippocampal sclerosis | Y | N | N | N | 74 | 39 | 113 | Lamotrigine, Levetiracetam |
| **10** | 54 | M | L | **N/A** | **N/A** | Hippocampal sclerosis | **N/A** | **N/A** | **N/A** | **N/A** | **N/A** | **N/A** | **N/A** | **N/A** |
| **11** | 26 | F | R | 12 | SPS | Hippocampal sclerosis | N | N | N | N | 75 | 44 | 119 | Lamotrigine, Levitericitam, Oxcarbazepine, Clobazam |
| **12** | 33 | F | L | **N/A** | **N/A** | **N/A** | **N/A** | **N/A** | **N/A** | **N/A** | 34 | 39 | 73 | **N/A** |

**TLE = temporal lobe epilepsy**

**CPS = complex partial seizure; GTCS = generalised tonic-clonic seizure; SPS = simple partial seizure**

**WAIS = Wechsler adult intelligence scale; VIQ = verbal IQ; PIQ = performance IQ; FSIQ = full scale IQ**

**N/A = Not available**
