# Supplementary material for: Lateralization of Temporal Lobe Epilepsy Based on Resting-State Functional Magnetic Resonance Imaging and Machine Learning
Source: Front Neurol. 2015 Aug 31;6:184. doi: 10.3389/fneur.2015.00184 (PMC4553409; doi:10.3389/fneur.2015.00184)
Supplement: Supplementary file 1 [file data_sheet_1.zip › 110661_TLE_suppl_2.docx]

**Supplementary Material 2.**

The clustering coefficient, characteristic path length, and small-world index (smallworldness) at six different network density levels. Each plot is for an individual subject.

**Supplementary Material 3.**

The mean values of the 11 global network metrics over all subjects (blue), the left TLE group (red) and the right TLE group (green) at different network density levels. The error bars indicate the standard error of the mean.
